# Association between air pollution and transplant outcomes in kidney transplant recipients: a systematic review and meta-analysis

**DOI:** 10.1093/ckj/sfaf222

**Published:** 2025-07-12

**Authors:** Mustafa Guldan, Lasin Ozbek, Derya Goksu Fidan, Ibrahim Gulmaliyev, Aladin Rustamov, Mehmet Kanbay

**Affiliations:** Department of Medicine, Koc University School of Medicine, Istanbul, Turkey; Department of Medicine, Koc University School of Medicine, Istanbul, Turkey; Department of Medicine, Koc University School of Medicine, Istanbul, Turkey; Department of Medicine, Koc University School of Medicine, Istanbul, Turkey; Department of Medicine, Koc University School of Medicine, Istanbul, Turkey; Department of Medicine, Section of Nephrology, Koc University School of Medicine, Istanbul, Turkey

**Keywords:** air pollution, allograft rejection, cardiovascular mortality, graft survival, kidney transplantation

## Abstract

**Background:**

Emerging evidence suggests that ambient air pollution may adversely affect long-term outcomes in kidney transplant recipients; however, quantitative estimates across clinical endpoints remain limited. This meta-analysis aimed to systematically evaluate the association between air pollution exposure and mortality, graft failure, and rejection risk in kidney transplant populations.

**Methods:**

A systematic database search was carried out across the databases of the Cochrane Library, Web of Science, Scopus, and PubMed until the 1 May 2025. Research that evaluated the impact of air pollution, particularly PM₂.₅, PM₁₀, NO₂, O₃, and other ambient pollutants, on graft survival in kidney transplant recipients were evaluated. Hazard ratios (HR) were extracted or recalculated for all-cause mortality, death-censored graft failure, and graft rejection per 10 µg/m³ increase in particulate matter concentration.

**Results:**

After screening 6209 records, a total of six studies involving populations of adult kidney transplant recipients from the USA, UK, South Korea, and Taiwan were included in the meta-analysis. Exposure to ambient air pollution was significantly associated with increased all-cause mortality among kidney transplant recipients [pooled HR 1.61; 95% confidence intervals (CI) 1.01–2.58], as well as higher risks of death-censored graft failure (HR 1.25; 95% CI 1.04–1.50) and graft rejection (HR 1.35; 95% CI 1.09–1.69) per 10 µg/m³ increment in particulate matter concentration. Substantial heterogeneity was observed across studies, particularly for mortality (*I*² = 99%) and graft rejection (*I*² = 91%). No significant associations were found between air pollution exposure and cardiovascular disease or coronary heart disease mortality.

**Conclusion:**

Ambient air pollution exposure is associated with increased risks of mortality, graft failure, and rejection in kidney transplant recipients, highlighting air pollution as a modifiable environmental risk factor that may have important implications for long-term transplant outcomes.

## INTRODUCTION

Air pollution, which is a well-established environmental risk factor for cardiovascular diseases, is frequently underrecognized in the context of chronic kidney disease (CKD), despite its capacity to impair glucose metabolism, alter blood pressure regulation, and induce oxidative stress and systemic inflammation [1, 2, 3]. Increasing evidence suggests that air pollution is an emerging yet overlooked contributor to CKD development and progression [3, 4]. While traditional risk factors such as diabetes, hypertension, and aging have been extensively studied, recent research has shifted focus toward environmental exposures, particularly air pollution, as significant drivers of renal dysfunction and adverse clinical outcomes.

Particulate matter (PM) with an aerodynamic diameter <2.5 µm (PM_2.5_) is a prevalent component of air pollution, consisting of a heterogeneous mixture of solid and liquid particles suspended in the atmosphere, with continuously varying chemical composition [5]. Fine PM_2.5_ has been recognized as a significant risk factor for numerous adverse health outcomes, including cardiovascular disease, diabetes, and all-cause mortality, primarily through its capacity to induce systemic inflammation. Emerging evidence also highlights PM_2.5_ as a major nephrotoxic agent. Due to its small size, PM_2.5_ can penetrate deep into the alveolar spaces, enter the systemic circulation, and reach the kidneys, where it activates inflammatory, oxidative, and fibrotic pathways that contribute to renal injury [6, 7]. Elevated PM_2.5_ exposure has been associated with incident kidney disease, reduced renal function, as evidenced by declines in estimated glomerular filtration rate (eGFR), higher rates of CKD and end-stage kidney disease (ESKD), and accelerated progression to kidney failure [7–9].

Vulnerable subpopulations, such as kidney transplant (KT) recipients, experience increased susceptibility due to triggering of the immune system by PM_2.5_, leading to inflammation. KT recipients may be particularly susceptible to the harmful effects of PM_2.5_ exposure due to their comorbidities and chronic immunosuppression [7, 8]. KT recipients are maintained on long-term immunosuppressive therapy, which compromises immune surveillance and heightens vulnerability to PM_2.5_-induced infections and malignancies. Furthermore, PM_2.5_ exposure may increase oxidative stress, contributing to the development of delayed graft function (DGF). Long-term exposure to PM_2.5_ can also impair allograft function by inducing systemic inflammatory responses, ultimately increasing the risk of DGF and solid organ rejection. Inhaled particulates may be filtered through the kidneys and directly damage renal tissue. Moreover, PM_2.5_ has been associated with insulin resistance, endothelial dysfunction, and systemic hypertension, which may further exacerbate renal injury and allograft deterioration [7, 9]. Several studies have reported associations between elevated PM_2.5_ exposure and higher rates of all-cause mortality, DGF, graft rejection, and graft failure among KT recipients [7, 10, 11].

Actually, results indicating an association between PM_2.5_ exposure and KT outcome are controversial. Given the limited and heterogeneous data on the relationship between PM_2.5_ levels and mortality or transplant-related outcomes in KT recipients, as well as the existing knowledge gap in this area, this systematic review and meta-analysis aimed to synthesize the current evidence on the impact of ambient air pollution on mortality, graft rejection, graft function, and graft survival in KT recipients. The findings from this study are intended to inform future research directions and support the development of clinical management strategies, public health interventions, and environmental policies to better protect this vulnerable population from preventable environmental hazards.

## MATERIALS AND METHODS

### Study design

This study was a systematic review and meta-analysis to investigate the association between ambient air pollution, particularly fine PM (PM_2.5_), and mortality and transplant outcomes in KT recipients. The study protocol was performed in accordance with the Preferred Reporting Items for Systematic Reviews and Meta-Analyses (PRISMA) guidelines [12]. The protocol was registered in the PROSPERO database under registration number CRD420251066564.

### Search strategy

A comprehensive literature search was conducted throughout May 2025 across multiple databases, including PubMed, Ovid MEDLINE, Scopus, Embase, Web of Science, and the Cochrane Library. The search strategy incorporated a combination of Medical Subject Headings (MeSH) and free-text terms related to (“kidney diseases” OR “renal diseases” OR “kidney transplantation” OR “kidney transplant” OR “renal transplantation”) AND (“air pollution” OR “air pollutant” OR particulate air pollutant” OR “particulate matter” OR “inhalable particles” OR “PM2.5” OR “PM10”) AND (“Outcomes” OR “survival” OR “kidney function” OR “kidney insufficiency” OR “kidney outcomes” OR “mortality” OR “All-cause mortality” OR “post-transplant morbidity” OR “Graft survival” OR “allograft survival” OR “long-term graft survival” OR “short-term graft survival” OR “graft outcomes” OR “allograft outcome” OR “graft failure” OR “graft dysfunction”). Detailed search strategies for each database and the complete list of search terms are provided in Supplementary Table S1

### Eligibility criteria

Eligible studies met the following criteria: (i) enrolled adult KT recipients; (ii) assessed exposure to elevated levels of ambient fine PM (PM2.5) or other air pollutants, measured either during the year preceding transplantation or as a time-dependent annual mean post-transplant; and (iii) reported outcomes including all-cause mortality, graft failure, DGF, death-censored graft failure, graft rejection, or allograft dysfunction. Both observational studies (cohort, case-control, and cross-sectional designs) and interventional studies were included, provided they reported quantitative associations between air pollution exposure and transplant-related outcomes.

Studies were excluded if they did not report relevant outcomes or lacked sufficient data for extraction. Additional exclusions included animal studies, *in vitro* studies, case reports, review articles, abstracts, editorials, commentaries, and letters without original data. Conference abstracts without full-text availability were also excluded. Furthermore, publications without an accessible full-text or an English-language version were not considered.

### Data extraction

Two independent reviewers screened the titles and abstracts, followed by a full-text review to determine eligibility based on predefined criteria. Discrepancies were resolved through consensus or consultation with a third independent reviewer. Data extraction was conducted using a standardized form to collect key study details, including the first author, year of publication, country, study design, sample size, participant characteristics (age and sex), type and dosage of air pollutants, methods of exposure assessment, parameters of kidney dysfunction investigated, reported outcomes, effect estimates, and adjusted confounders.

### Quality assessment

The quality of the studies included was assessed independently by two reviewers using the Newcastle–Ottawa Scale (NOS). A third reviewer independently assisted in resolving any disagreements. The NOS evaluates the domains of selection, comparability, and outcome, with a maximum score of nine points assigned to cohort and case-control studies. Studies scoring seven or more were considered high quality, those scoring between four and six as moderate quality, and those with scores of three or less as poor quality (Supplementary Table S2).

### Statistical analysis

For each included study, hazard ratios (HRs) and corresponding 95% confidence intervals (CIs) were extracted for the association between ambient air pollution exposure and clinical outcomes of interest, including all-cause mortality, death-censored graft failure, and graft rejection. Where studies reported effect estimates per 1 µg/m³ increment, values were recalculated to reflect a standardized 10 µg/m³ increase to facilitate comparability across studies. Odds ratios (ORs) reported for graft rejection were considered approximate HRs under the rare outcome assumption. Random-effects meta-analyses were performed using the DerSimonian and Laird method to pool effect estimates, accounting for between-study variability. Statistical heterogeneity was assessed using the *I*² statistic, Tau², and Cochran's *Q* test, with *I*² values >50% considered indicative of substantial heterogeneity. All analyses were conducted using Review Manager version 5.3 (The Cochrane Collaboration), and statistical significance was set at a two-sided *P* value <.05.

## RESULTS

After screening 6209 records, six studies met the eligibility criteria and were included in the meta-analysis [5, 7, 10, 13–15]. Hence, this meta-analysis included six retrospective cohort studies evaluating the association between ambient air pollution exposure and clinical outcomes in adult KT recipients. The studies were conducted across multiple regions, including the USA, UK, and South Korea, with sample sizes ranging from 1532 to >93 000 recipients. All participants had functioning renal allografts at baseline. Air pollution exposure was primarily characterized by PM [≤2.5 µm (PM₂.₅) or ≤10 µm (PM₁₀)], nitrogen dioxide (NO₂), and ozone (O₃), with exposure assessments derived from various sources including satellite-based hybrid models, land-use regression traffic models, and governmental air quality monitoring networks. Exposure assignment was typically based on residential ZIP code or postal code at the time of transplantation, although geographic resolution varied across studies (Table 1). The main outcomes assessed included all-cause mortality, death-censored graft failure, DGF, and acute or biopsy-proven rejection episodes. Cause-specific mortality was not assessed since it was not reported in all the studies screened; out of the studies screened, the ones that reported cause-specific mortality are presented on Supplementary Table S7. For articles that reported cause-specific mortality, the only one that reported infectious disease or pulmonary-disease related mortality was Kim *et al.*, which found no significant associations with PM₁₀ exposure (adjusted HR 1.05, 95% CI 0.92–1.20). Most studies applied multivariable adjustment for key clinical and demographic confounders, including recipient age, sex, race, body mass index, comorbidities, dialysis duration, donor type, ischemia time, and socioeconomic status. Follow-up durations across studies ranged from a median of 5.5 to nearly 15 years, providing substantial longitudinal data for outcome assessment (Table 2).

**Table 1:** Pollution measurement methods and populations of the studies analyzed.

Study	Design	Country	Studied population (*N*)	Renal status at baseline	Pollution parameters	Measurement method	Exposure assignment
Feng 2021 [7]	Registry-based retrospective cohort	USA	≈87 000 adult KT recipients (UNOS/OPTN 2010–2016)	Functioning renal allografts	Annual PM₂.₅ (µg/m³)	NASA satellite + EPA ground-monitor fusion model	Mean annual PM₂.₅ linked to residential ZIP at time of transplant
Spencer-Hwang 2011 [13]	Retrospective cohort	USA (California)	10 658 KT recipients 1988–2006	Functioning grafts	O₃ (ppb), PM₁₀ (µg/m³)	County ambient-monitor network	Annual pollutant averages assigned by transplant-county
Chang 2021 [9]	Retrospective cohort	USA	87 094 KT recipients 2004–16	Functioning grafts	Annual PM₂.₅	Satellite–monitor hybrid, 1-km model	ZIP code PM₂.₅ at transplant (time-varying sensitivity)
Dehom 2021 [10]	Retrospective cohort	USA	93 857 renal-TX recipients 2001–15	Functioning grafts (non-smokers)	Annual PM₂.₅	US EPA monitor network	Year-specific mean PM₂.₅ mapped to residential ZIP (time-varying)
Pierotti 2018 [15]	Retrospective cohort	UK	20 077 solid-organ TX recipients (≈60 % kidney) 1995–2015	Functioning grafts	NO₂ (µg/m³)	Land-use-regression traffic model	Road-network NO₂ at postcode centroid
Kim 2021 [14]	Single-center retrospective cohort	South Korea	1 532 KT recipients 2001–15	Functioning grafts	PM₁₀ (µg/m³)	National monitoring stations	City-level annual PM₁₀ (hospital ≈ residence)
Lin 2020 [6]	Registry-based retrospective cohort	Taiwan	6 628 CKD stage 3–5 patients 2003–15	Non-TX CKD	PM₂.₅	Satellite spatiotemporal model (1 km)	Baseline 1-y mean PM₂.₅ at geocoded home address

UNOS, United Network for Organ Sharing OPTN, Organ Procurement and Transplantation Network TXl transplantation NASA, National Aeronautics and Space Administration US EPA, US Environmental Protection Agency ZIPl Zone Improvement Plan.

**Table 2:** Outcomes, definitions, follow-up duration, and adjustment factors of the articles analyzed.

Study	Outcomes and definitions	Core adjustment factors	Key results (direction + estimate)	Follow-up duration (years)
Feng 2021 [7]	• Delayed graft function = dialysis ≤7 d post-TX • 1-year biopsy-confirmed rejection • Death-censored graft failure (DCGF) • All-cause death	Age, sex, race, BMI, dialysis vintage, donor and cold-ischemia times, smoking, ZIP-SES	+10 µg/m³ PM₂.₅ → OR 1.59 (DGF), OR 1.31 (1-y rejection); HR 1.14 (DCGF), HR 1.15 (death)	Median 5.5
Spencer-Hwang 2011 [13]	Fatal CHD (ICD-9 410–414 on death certification)	Age, sex, race, transplant year	+10 ppb O₃ → RR 1.34 fatal CHD; PM₁₀ ns	≈7
Chang 2021 [9]	Acute rejection, DCGF, all-cause death	Age, sex, race, BMI, donor factors, dialysis time, ZIP-SES	PM₂.₅ IQR (∼10 µg/m³) ↑ HR 1.14 (DCGF) & HR 1.18 (death); rejection similar	Median 6.0 (IQR 3.9–8.9)
Dehom 2021 [10]	All-cause, total CVD, CHD death (National Death Index)	Age, sex, diabetes, hypertension, BMI, donor type, ZIP income	+10 µg/m³ PM₂.₅ → HR 3.45 (all); HR 2.38 (CVD); HR 3.10 (CHD); Blacks HR 4.09 (all)	Median 14.9
Pierotti 2018 [15]	Transplant failure = graft loss or death	Age, sex, organ, year, area deprivation	NO₂ quintiles → no significant change in failure risk	∼10
Kim 2021 [14]	Biopsy-proven rejection, DCGF, all-cause death	Age, sex, diabetes, HTN, eGFR, donor type, BMI, HLA mismatch	+1 µg/m³ PM₁₀ → HR 1.05 (DCGF) & HR 1.09 (death); HR 1.05 (rejection)	Median 6.31
Lin 2020 [6]	KFRT, all-cause death	Age, sex, BP, diabetes, eGFR, income, urbanicity	+7.8 µg/m³ PM₂.₅ → HR 1.19 (KFRT); mortality neutral	Mean 4.3

CHD, chronic heart disease, DCGF, death-censored graft failure, ICD: International Classification of Disease KFRT, kidney failure requiring replacement therapy; ZIP-SES: Zone Improvement Plan—Socioeconomic Index, HTN: hypertension nephropathy HLA, human leukocyte antigens BP, blood pressure RR, relative risk (ratio of probability of an event between two groups); ns, not significant.

### All-cause mortality

Five studies provided findings on the outcome of all-cause mortality [7, 9, 10, 13, 14]. Exposure to ambient air pollution was significantly associated with increased all-cause mortality, yielding a pooled HR of 1.61 (95% CI: 1.01–2.58; *Z* = 1.99, *P* = .05), with substantial heterogeneity observed (*I*² = 99%, Tau² = 0.28, *P* < .00001) (Fig. 2). Individual study estimates varied, with Chang *et al.* [9] reporting an HR of 1.21 [95% CI: 1.14–1.28; log(HR) = 0.1906, SE = 0.0293], Dehom *et al.* [10] reporting 3.45 (95% CI: 3.11–3.82; log(HR) = 1.2380, SE = 0.0523], and Feng *et al.* [7] reporting 1.15 [95% CI: 1.07–1.23; log(HR) = 0.1398, SE = 0.0355]. Kim *et al.* [14] originally reported HR per 1 µg/m³ (linear) increase in ambient air pollution (HR = 1.090, 95% CI: 1.043–1.140); this estimate was recalculated to reflect a 10 µg/m³ increment, yielding an HR of 2.3657 [log(HR) = 0.8610, SE = 0.2269]. Spencer-Hwang *et al.* [13] reported an HR of 1.02 [95% CI: 0.96–1.09; log(HR) = 0.0198, SE = 0.0324]. The observed heterogeneity likely reflects variations in study populations, exposure levels, particulate composition, and study methodologies.

**Figure 1:**
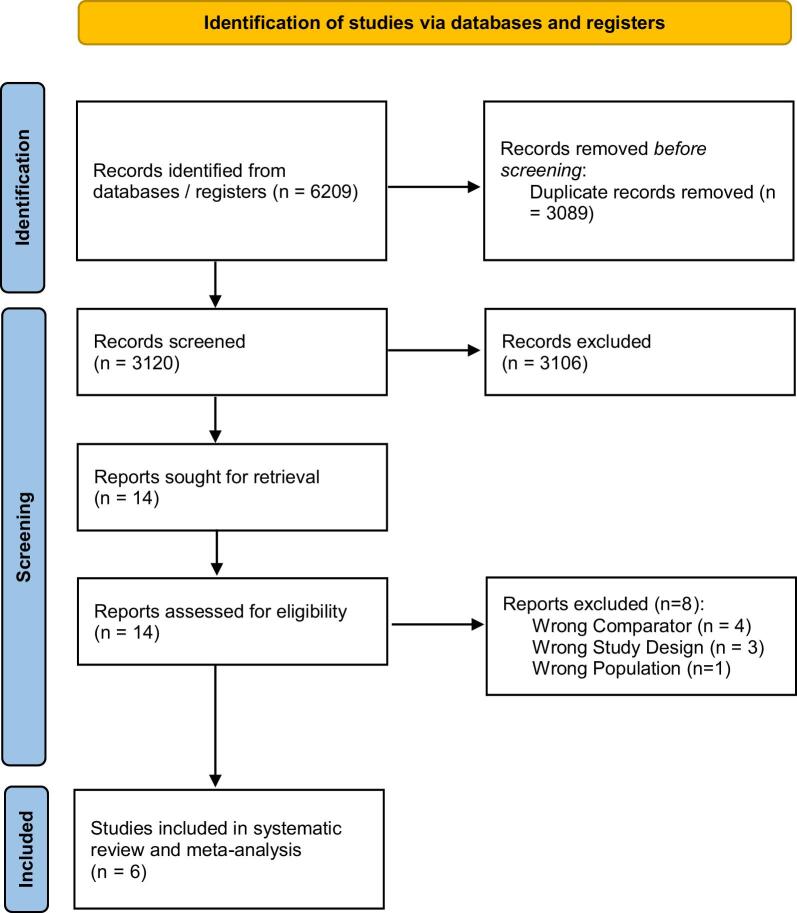
Flow diagram of the study selection process.

**Figure 2:**
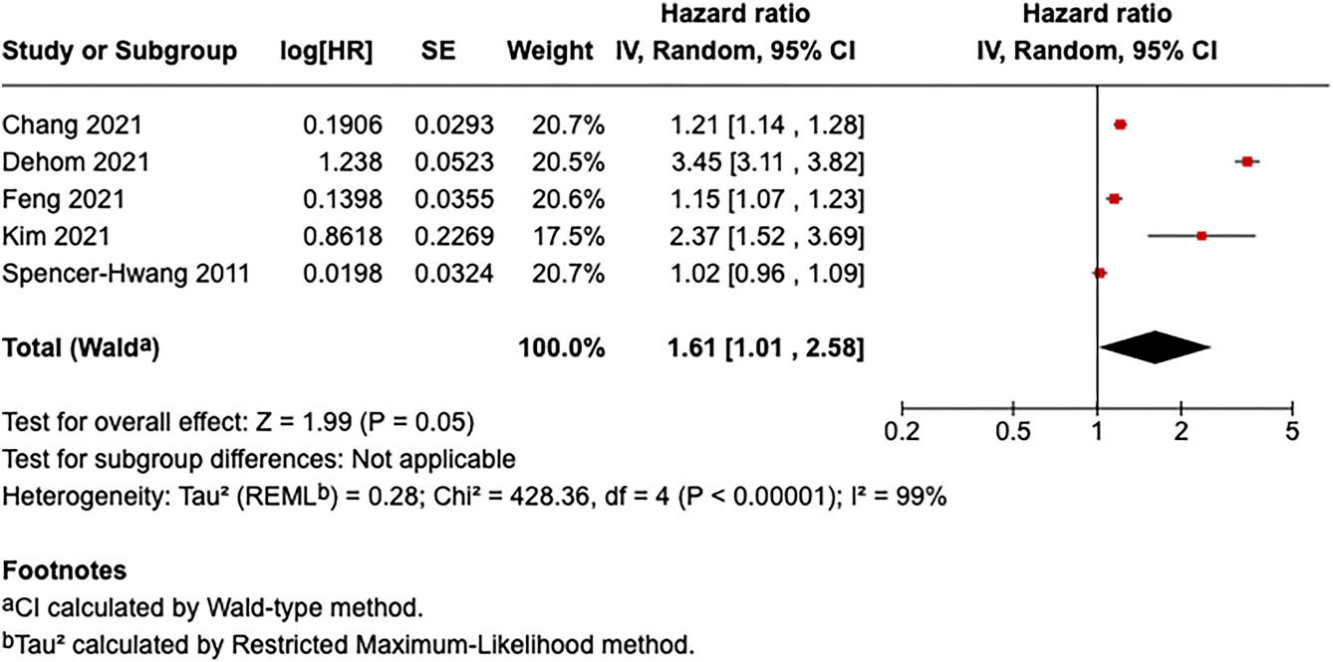
Association between air pollution exposure and all-cause mortality.

### Death-censored kidney graft failure

**Figure 3:**
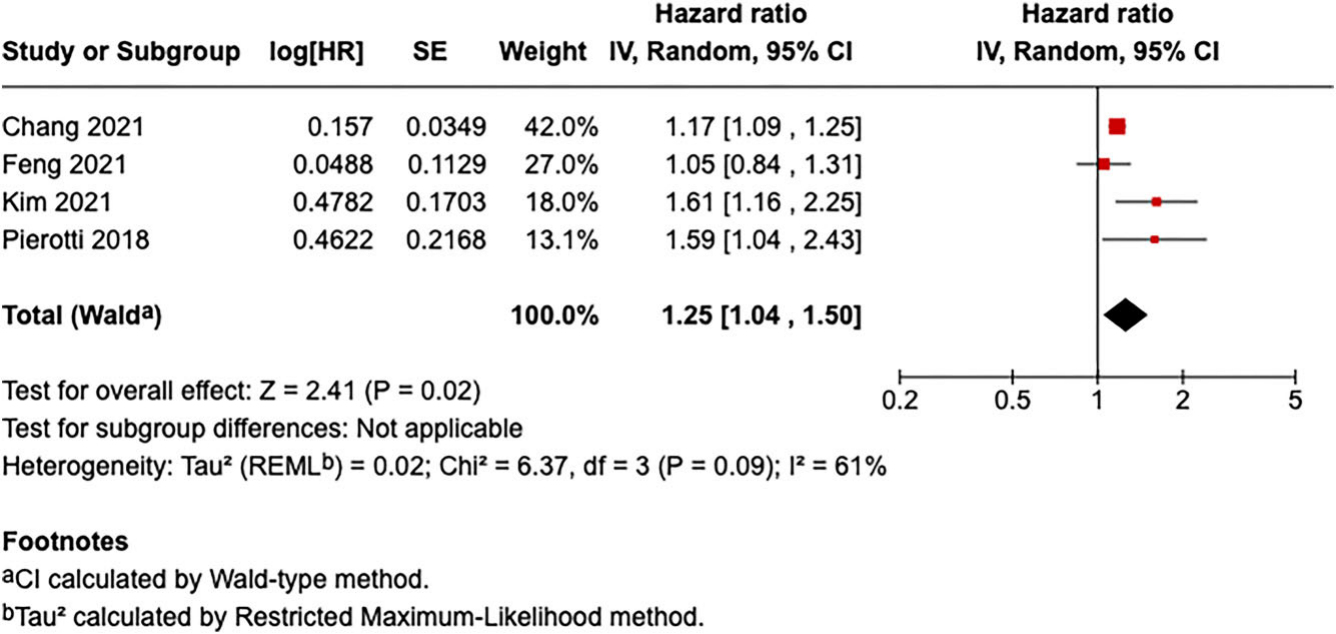
Association between ambient air pollution exposure and risk of death-censored kidney graft failure.

Four studies were included in the meta-analysis of the association between air pollution and death-censored renal allograft failure [7, 9, 14, 15]. Exposure to air pollution was significantly associated with an increased risk of death-censored renal allograft failure (Fig. 3). The pooled HR was 1.25 (95% CI: 1.04–1.50; *Z* = 2.41; *P* = .02), indicating a 25% increased risk per 10 µg/m³ increment in ambient air pollution concentration. Heterogeneity among studies was moderate (*I*² = 61%, Tau² = 0.02, *P* = .09). Chang *et al.* [9] reported an HR of 1.17 [95% CI: 1.09–1.25; log(HR) = 0.1570, SE = 0.0349], while Feng *et al.* [7] reported a smaller and non-significant association [HR = 1.05, 95% CI: 0.84–1.31; log(HR) = 0.0488, SE = 0.1129]. Kim *et al.* [14] originally reported an HR of 1.049 (95% CI: 1.014–1.084) per 1 µg/m³ increase, which was recalculated for a 10 µg/m³ increase, yielding HR = 1.6129 [log(HR) = 0.4780, SE = 0.1700]. Similarly, Pierotti *et al.* [15] originally reported an HR of 1.26 (95% CI: 1.02–1.56) per 5 µg/m³ increase, which was recalculated for 10 µg/m³, resulting in HR = 1.5871 [log(HR) = 0.4622, SE = 0.2168]. The consistent positive associations across most studies support the detrimental role of ambient air pollution in compromising long-term graft survival in KT recipients.

### Graft rejection

**Figure 4:**
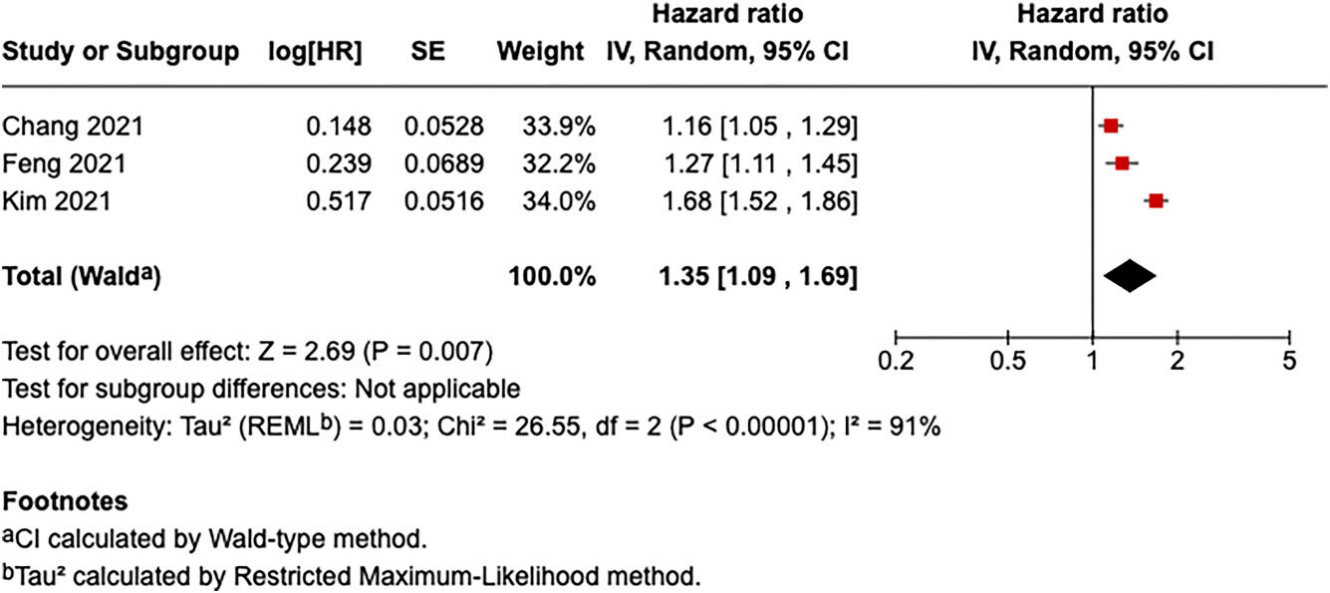
Association between air pollution exposure and renal graft rejection.

Three studies were included to evaluate the association between ambient air pollution exposure and the risk of graft rejection (Fig. 4, Supplementary Table S3) (7, 9, 14). The pooled analysis demonstrated a significant association between increased ambient air pollution exposure and graft rejection (HR: 1.35, 95% CI: 1.09–1.69; *P* = .007). Substantial heterogeneity was observed across studies (*I*² = 91%, *P* < .00001). Chang *et al.* [9] reported a HR for graft rejection per 10 µg/m³ increment in ambient air pollution concentration, yielding an HR of 1.16 (95% CI: 1.05–1.29). Feng *et al.* [7] provided data on the OR for 1-year acute rejection, reporting an OR of 1.27 (95% CI: 1.11–1.45). Given the lack of time-to-event data, and based on the assumption of rare outcomes and similar biological plausibility, the OR was considered an approximate HR for this meta-analysis. Kim *et al.* [14] presented an HR for each 1 µg/m³ increment of ambient air pollution exposure, which was recalculated to reflect a 10 µg/m³ increment, resulting in an HR of 1.68 (95% CI: 1.52–1.86).

The pooled effect sizes for cardiovascular disease (CVD) risk and coronary heart disease (CHD) fatality were not statistically significant in our analyses (Supplementary Fig. S1). The definitions of cardiovascular outcomes are defined in Supplementary Table S4.

## DISCUSSION

In this meta-analysis, we demonstrate that exposure to ambient air pollution is significantly associated with adverse outcomes in KT recipients. Our pooled estimates show that higher air pollution exposure correlates with increased all-cause mortality, with a HR of 1.61, despite notable heterogeneity across studies, likely reflecting differences in population characteristics, exposure assessment methods, and geographic variability. Importantly, beyond mortality, we also observed a significant association between air pollution exposure and both death-censored graft failure and graft rejection, with HRs of 1.25 and 1.35, respectively. These consistent findings across multiple studies support the hypothesis that ambient air pollution exerts deleterious effects not only on patient survival but also directly on allograft function and immunologic stability. Interestingly, no significant association was observed with cardiovascular and CHD mortality, suggesting that non-cardiovascular mechanisms, such as immune modulation, systemic inflammation, and endothelial dysfunction, may play a more central role in mediating these adverse transplant outcomes. Collectively, these findings underscore the relevance of air pollution as a modifiable, yet largely overlooked, environmental risk factor in KT, warranting further mechanistic studies and potentially informing future risk stratification and preventive strategies in this high-risk population.

The included studies in this meta-analysis demonstrate both the growing interest and the evolving methodological approaches used to investigate air pollution exposure in KT populations. Despite differences in study design, geographic setting, and pollution assessment techniques, the majority of studies applied reasonably advanced exposure assignment methods, incorporating satellite-based models, ground-monitor fusion data, and geographic linkage to residential ZIP or postal codes. Importantly, although most studies examined PM₂.₅ as the primary pollutant of interest, several incorporated alternative or additional exposures, including PM₁₀, ozone, and nitrogen dioxide, allowing for some cross-pollutant comparisons. For example, in one of the included large-scale studies focusing on air pollution in KT recipients, Spencer-Hwang *et al.* analyzed a cohort of 32 239 nonsmoking adult transplant recipients from the US Renal Data System, assigning long-term exposure to ozone (O₃) and PM₁₀ based on proximity to EPA monitoring stations. Over a 7-year follow-up, elevated ambient ozone exposure was significantly associated with increased CHD mortality, with each 10-ppb (parts per billion) increment in O₃ linked to a 34–35% higher risk of fatal CHD (RR 1.34–1.35, depending on model specification) [13]. In contrast, no independent association was observed between PM₁₀ exposure and either CHD or all-cause mortality, suggesting potential pollutant-specific vascular effects. Remarkably, these findings highlight not only the plausibility of pollutant heterogeneity in mediating cardiovascular versus graft-related outcomes but also underscore the importance of evaluating multiple pollutant classes when assessing environmental risks in transplant populations.

Adjustment for relevant clinical covariates was generally robust across studies, with consistent incorporation of key transplant-specific and demographic variables, although variability remained in the granularity of socioeconomic and immunologic adjustment factors, which could be an issue to be addressed in future studies. The heterogeneity in sample sizes, ranging from large registry cohorts to smaller single-center analyses, reflects both the strengths and limitations of the current evidence base: while registry-based studies enhance generalizability, smaller cohorts may offer greater clinical detail. Collectively, these studies provide a broad yet coherent foundation that strengthens the biological plausibility of the observed associations, while also highlighting areas where future investigations could improve exposure precision, incorporate time-varying exposure metrics, and better capture center-level or regional differences in transplant practice.

### Air pollution, chronic kidney disease occurrence, and outcomes

Air pollution, particularly fine PM (PM₂.₅), has been increasingly recognized as a significant environmental risk factor for CKD. Recent studies have demonstrated that long-term exposure to PM₂.₅ is associated with heightened risks of CKD incidence, progression, and mortality. For instance, a meta-analysis encompassing 14 cohort studies and nearly 8 million participants revealed that each 10 μg/m³ increase in PM₂.₅ concentration corresponds to a 31% higher risk of both CKD incidence and prevalence[16]. The association with ESKD incidence was suggestive but not conclusive, with an adjusted OR of 1.16 (95% CI: 1.00 to 1.36). Similarly, another systematic review reported a 42% increased risk of CKD per 10 μg/m³ increment in PM₂.₅ exposure[17, 18]. Furthermore, a large-scale cohort study involving >2.4 million US veterans found that a 10 μg/m³ rise in PM₂.₅ levels was associated with a 27% increased risk of incident CKD, a 28% higher likelihood of significant eGFR decline, and a 26% elevated risk of ESKD [19]. These associations persisted even at PM₂.₅ concentrations below current regulatory standards, suggesting that existing air quality guidelines may not sufficiently protect kidney health. Air pollution, particularly fine PM (PM₂.₅), has been implicated not only in the increased incidence and prevalence of CKD but also in adverse outcomes such as accelerated progression to ESKD, heightened mortality rates, and increased susceptibility to acute kidney injury (AKI) [20].

### Mechanisms of air pollution-induced renal injury: implications for transplant recipients

The nephrotoxic effects of air pollution are mediated through several interrelated mechanisms: inhaled pollutants such as PM₂.₅ and nitrogen dioxide (NO₂) can translocate into the systemic circulation, eliciting oxidative stress and systemic inflammation, which in turn compromise renal endothelial function and promote glomerular and tubular injury [3]. Additionally, these pollutants can induce hypertension and insulin resistance, further exacerbating renal damage. Experimental studies have also demonstrated that PM₂.₅ exposure activates autophagic pathways in renal tubular epithelial cells, leading to cellular apoptosis and fibrosis [21].

In the context of KT, these mechanisms may have heightened significance, yet they are largely unexplored. Transplant recipients are often on chronic immunosuppressive therapy, which can exacerbate oxidative stress and impair immune responses. This immunosuppressed state may render the allograft more susceptible to the deleterious effects of air pollution, potentially leading to increased risks of acute rejection, chronic allograft dysfunction, and reduced graft survival. Furthermore, systemic inflammation induced by air pollutants may amplify alloimmune responses, further compromising graft integrity. Understanding these mechanisms underscores the importance of mitigating air pollution exposure in KT recipients to preserve allograft function and improve long-term outcomes.

### Integrated clinical practice and policy implications

Given the findings of this meta-analysis suggesting associations between air pollution exposure and key transplant outcomes, including all-cause mortality, death-censored graft failure, and acute rejection, environmental exposure may represent a previously underrecognized yet modifiable contributor to long-term KT care. These results support the need to incorporate environmental risk assessment into the longitudinal care of KT recipients, particularly for patients residing in high-exposure urban or industrial regions. In clinical practice, transplant programs may consider integrating environmental exposure assessment into routine risk stratification, potentially incorporating patient residential air quality data as part of pre- and post-transplant evaluations. For recipients residing in areas with persistently high levels of air pollution, more frequent monitoring of graft function, individualized immunosuppressive drug level assessments, and earlier screening for subclinical rejection or vascular complications may be warranted. Moreover, personalized counseling on behavioral strategies to minimize pollutant exposure, such as the use of air filtration systems, avoidance of outdoor activities during high pollution days, and smoking cessation, may help attenuate cumulative exposure.

Furthermore, targeted public health interventions and policy efforts aimed at reducing ambient air pollution may yield meaningful benefits for transplant populations. From a health policy perspective, the growing body of evidence linking air pollution to transplant outcomes may justify the development of targeted environmental interventions for vulnerable populations, including the creation of registries linking transplant cohorts to geospatial pollution data, or even financial support for housing relocation to cleaner environments in selected high-risk patients. Additionally, environmental health equity considerations should be addressed, as many transplant recipients reside in socioeconomically disadvantaged areas with disproportionately higher pollution burdens. Public health policies and environmental health initiatives that promote cleaner residential environments or provide incentives for transplant recipients to reside in areas with lower air pollution levels may offer an opportunity to mitigate environmentally driven risks to graft function and patient survival.

### Future directions

Future research should prioritize large-scale, prospective, multicenter studies with standardized exposure assessment methods to better characterize the dose–response relationship between air pollution and transplant outcomes. Additionally, mechanistic studies are needed to elucidate the pathways by which air pollution contributes to graft injury, including potential effects on systemic inflammation, endothelial dysfunction, alloimmune activation, and immunosuppressive pharmacokinetics. Importantly, studies addressing vulnerable subgroups, such as recipients with pre-existing cardiovascular or pulmonary comorbidities, may help refine individualized risk stratification models. In addition, once studies are released exploring pollution levels on different countries or continents, a meta-analysis can be carried out to assess the effects of pollution on kidney transplantation outcomes across different nations.

### Limitations

Several limitations of this meta-analysis warrant consideration. First, the number of available studies remains relatively small, limiting the precision of pooled estimates and precluding robust subgroup or sensitivity analyses. Second, substantial heterogeneity was observed across studies, likely reflecting differences in study design, exposure assessment methodologies (e.g. varying air pollution metrics, spatial resolution, and exposure windows), population demographics, and immunosuppressive regimens. Third, most studies were observational and thus susceptible to residual confounding from unmeasured variables such as socioeconomic status, comorbid conditions, medication adherence, and center-specific transplant practices. Finally, limited data were available on pollutant subtypes beyond PM₂.₅ (e.g. NO₂, ozone), restricting our ability to assess differential pollutant-specific effects on transplant outcomes.

### Strengths

This meta-analysis has several notable strengths. First, it represents the most comprehensive quantitative synthesis to date evaluating the association between ambient air pollution exposure and a range of clinically relevant outcomes in KT recipients. Second, this meta-analysis incorporates multiple clinically meaningful endpoints, including death-censored graft failure and biopsy-proven acute rejection, allowing for a more granular assessment of both patient and graft outcomes. Third, the included studies applied generally robust multivariable adjustment for key transplant-specific covariates, such as dialysis vintage, donor characteristics, ischemia time, comorbidities, and socioeconomic status, thereby minimizing confounding from well-established prognostic factors. Finally, this analysis is strengthened by the inclusion of multiple pollutant classes (PM₂.₅, PM₁₀, O₃, and NO₂), which offers important early insights into potential pollutant-specific risk patterns within the transplant population, a dimension that has been largely underexplored in prior environmental health studies. By integrating data across diverse geographic regions, including North America, Europe, and Asia, and capturing both large registry-based cohorts and smaller, high-resolution center-level studies, this analysis enhances the generalizability of findings across various healthcare settings and environmental exposure profiles.

## CONCLUSION

In summary, this meta-analysis provides the first quantitative synthesis demonstrating that ambient air pollution is independently associated with increased mortality, graft failure, and rejection risk in KT recipients. While substantial heterogeneity remains, the consistency of observed associations across studies highlights air pollution as a clinically relevant, potentially modifiable environmental risk factor. These findings underscore an urgent need for greater attention to environmental determinants of transplant outcomes, both in clinical practice and in public health policy. Despite the accumulating evidence linking air pollution to adverse renal and transplant outcomes, its specific impact on the KT population remains underexplored. Given the heightened vulnerability of transplant recipients due to immunosuppression and existing comorbidities, there is a pressing need for targeted research to elucidate the extent to which environmental pollutants influence graft survival and patient mortality in this group.

## Supplementary Material

sfaf222_Supplemental_Files

## Data Availability

Our paper has no associated data.

## Notes

PROSPERO registration number: CRD420251066564
