# Supplementary material for: Association between air pollution and transplant outcomes in kidney transplant recipients: a systematic review and meta-analysis
Source: Clin Kidney J. 2025 Jul 12;18(8):sfaf222. doi: 10.1093/ckj/sfaf222 (PMC12451696; doi:10.1093/ckj/sfaf222)
Supplement: sfaf222_Supplemental_Files [file sfaf222_Supplemental_Files.zip › 819 Supplementary Tables for the Revision.docx]

**Supplementary Table S5.** Population characteristics in the studies analyzed.

| **Study** | **n** | **Mean age (years)** | **Women (%)** | **Dialysis duration (years)** | **Living donor (%)** | **CNI-based IS (%)** |
| --- | --- | --- | --- | --- | --- | --- |
| Moon 2023 | 232 | 46 ± 11 | 39 | — | 100 | — |
| Feng 2021 | 87 233 | 54 (IQR 42 – 63) | 39 | 3.2 (high PM) / 2.3 (low PM) | 34 | 86 |
| Kim 2021 | 1 146 | 45 ± 13 | 40 | 3.8 ± 3.9 | 68 | 93 |
| Dehom 2021 | 1 146 | 50 ± 12 | 38 | 3.5 ± 2.7 | 40 | 90 |
| Spencer-Hwang 2011 | 5 059 | 49 ± 14 | 42 | 3.1 ± 2.5 | 33 | 88 |
| Pierotti 2022 | 6 174 | 48 (IQR 38 – 58) | 40 | 3.0 ± 2.4 | 35 | 87 |

**Supplementary Table S6.** Characteristics of the exposure windows.

| **Study** | **Pre-KT exposure window** | **Post-KT exposure window** | **Metric** |
| --- | --- | --- | --- |
| Moon 2023 | N/A | First post-op year | Annual mean PM₂․₅ |
| Feng 2021 | Calendar year of KT† | Same as pre | Annual mean PM₂․₅ |
| Kim 2021 | Entire dialysis period (mean 3.8 y) | Annual mean thereafter | PM₁₀ |
| Dehom 2021 | 3-yr moving average centred on KT yr | Rolling 3-yr average | PM₂․₅ |
| Spencer-Hwang 2011 | Baseline annual mean | Updated annually | PM₁₀ |
| Pierotti 2022 | Not separated; tied to residential history | Same metric annually | Road-NOx index |

† No separate pre- vs post-exposure; year of KT only.

**Supplementary Table S7.** Cause-specific mortality in the studies screened.

| **Study** | **Exposure comparison** | **Total deaths** | **Cardiovascular deaths (%)** | **Other causes (%)** |
| --- | --- | --- | --- | --- |
| Dehom 2021 | Highest vs lowest PM₂․₅ tertile | 115 | 54 (47 %) | 61 (53 %) |
| Spencer-Hwang 2011 | ≥90th vs ≤10th percentile PM₁₀ | 97 | 37 (38 %) | 60 (62 %) |
| Feng 2021 | Per 10 µg m⁻³ higher PM₂․₅ | 2 402 | 979 (41 %) | 1 423 (59 %) |
