# Supplementary material for: Association between air pollution and transplant outcomes in kidney transplant recipients: a systematic review and meta-analysis
Source: Clin Kidney J. 2025 Jul 12;18(8):sfaf222. doi: 10.1093/ckj/sfaf222 (PMC12451696; doi:10.1093/ckj/sfaf222)
Supplement: sfaf222_Supplemental_Files [file sfaf222_Supplemental_Files.zip › Supplementary Appendix (1).docx]

**Supplementary Table S1**. Search strategy and keywords for systematic database search

| **Total Records: n= 6209** |
| --- |
| - After Delete of Duplicate Records (EndNote): **n=3582** - After Delete of Duplicate Records (Covidence and manual identification): **n=3120** (n=460 duplicates identified by Covidence, n=2 duplicates identified manually) |
| **PubMed (n=1137)** |
| **4** (("Kidney Transplantation"[Title/Abstract] OR "Renal Transplantation"[Title/Abstract] OR "Kidney Transplant"[Title/Abstract] OR "Renal Transplant"[Title/Abstract] OR "Kidney Allograft"[Title/Abstract] OR "Renal Allograft"[Title/Abstract] OR "Kidney Graft"[Title/Abstract] OR "Renal Graft"[Title/Abstract] OR "Kidney Allograft Transplantation"[Title/Abstract] OR "Kidney Grafting"[Title/Abstract] OR "Renal Grafting"[Title/Abstract] OR "Kidney Retransplantation"[Title/Abstract] OR "Kidney Transplant Recipient"[Title/Abstract] OR "Renal Transplant Recipient"[Title/Abstract] OR "Kidney Recipient"[Title/Abstract] OR "Renal Recipient"[Title/Abstract]) AND ("Air Pollution"[Title/Abstract] OR "Outdoor Air Pollution"[Title/Abstract] OR "Traffic-Related Air Pollution"[Title/Abstract] OR "Air Quality"[Title/Abstract] OR "Polluted Air"[Title/Abstract] OR "Ambient Air Pollution"[Title/Abstract] OR "Atmospheric Pollution"[Title/Abstract] OR "Environmental Pollution"[Title/Abstract] OR "Environmental Exposure"[Title/Abstract] OR "Airborne Pollutants"[Title/Abstract] OR "Environmental Pollutants"[Title/Abstract] OR "Air Pollutants"[Title/Abstract] OR "Particulate Matter"[Title/Abstract] OR "Particulate Air Pollutants"[Title/Abstract] OR "Inhalable Particles"[Title/Abstract] OR "Fine Particulate Matter"[Title/Abstract] OR "Fine Particles"[Title/Abstract] OR "Ultrafine Particles"[Title/Abstract] OR "PM"[Title/Abstract] OR "PM0.1"[Title/Abstract] OR "PM1"[Title/Abstract] OR "PM2.5"[Title/Abstract] OR "PM10"[Title/Abstract] OR "PMcoarse"[Title/Abstract] OR "Coarse Particles"[Title/Abstract] OR "Black Carbon"[Title/Abstract] OR "BC"[Title/Abstract] OR "Ozone"[Title/Abstract] OR "O3"[Title/Abstract] OR "Carbon Monoxide"[Title/Abstract] OR "CO"[Title/Abstract] OR "Nitrogen Oxides"[Title/Abstract] OR "NOx"[Title/Abstract] OR "Nitrogen Dioxide"[Title/Abstract] OR "NO2"[Title/Abstract] OR "Sulfur Dioxide"[Title/Abstract] OR "SO2"[Title/Abstract])) AND ("Graft Survival"[Title/Abstract] OR "Allograft Survival"[Title/Abstract] OR "Kidney Graft Survival"[Title/Abstract] OR "Renal Graft Survival"[Title/Abstract] OR "Transplant Survival"[Title/Abstract] OR "long-term graft survival"[Title/Abstract] OR "short-term graft survival"[Title/Abstract] OR "survival"[Title/Abstract] OR "prognosis"[Title/Abstract] OR "outcome"[Title/Abstract] OR "Death-Censored Graft Failure"[Title/Abstract] OR "DCGF"[Title/Abstract] OR "Graft Failure"[Title/Abstract] OR "Kidney Transplant Failure"[Title/Abstract] OR "Renal Transplant Failure"[Title/Abstract] OR "Graft Loss"[Title/Abstract] OR "Graft Dysfunction"[Title/Abstract] OR "Renal Graft Dysfunction"[Title/Abstract] OR "Kidney Graft Dysfunction"[Title/Abstract] OR "Delayed Graft Function"[Title/Abstract] OR "DGF"[Title/Abstract] OR "Graft Function"[Title/Abstract] OR "Renal Function"[Title/Abstract] OR "Kidney Function"[Title/Abstract] OR "Estimated Glomerular Filtration Rate"[Title/Abstract] OR "eGFR"[Title/Abstract] OR "Glomerular Filtration Rate"[Title/Abstract] OR "GFR"[Title/Abstract] OR "Creatinine Clearance Rate"[Title/Abstract] OR "Serum Creatinine"[Title/Abstract] OR "Plasma Creatinine"[Title/Abstract] OR "Renal Insufficiency"[Title/Abstract] OR "Kidney Insufficiency"[Title/Abstract] OR "Proteinuria"[Title/Abstract] OR "Albuminuria"[Title/Abstract] OR "Urinary Protein-to-Creatinine Ratio"[Title/Abstract] OR "Graft Rejection"[Title/Abstract] OR "Allograft Rejection"[Title/Abstract] OR "Transplant Rejection"[Title/Abstract] OR "Organ Rejection"[Title/Abstract] OR "Acute Rejection"[Title/Abstract] OR "Chronic Rejection"[Title/Abstract] OR "Chronic Allograft Nephropathy"[Title/Abstract] OR "Chronic Antibody-Mediated Rejection"[Title/Abstract] OR "Biopsy-Proven Rejection"[Title/Abstract] OR "BPR"[Title/Abstract] OR "Morbidity"[Title/Abstract] OR "Post- Transplant Morbidity"[Title/Abstract] OR "Mortality"[Title/Abstract] OR "All-Cause Mortality"[Title/Abstract] OR "Death"[Title/Abstract] OR "Survival Rate"[Title/Abstract] OR "Infection"[Title/Abstract] OR "Sepsis"[Title/Abstract] OR "Adverse Outcomes"[Title/Abstract] OR "Transplant Complications"[Title/Abstract] OR "Short-Term Outcomes"[Title/Abstract] OR "Long-Term Outcomes"[Title/Abstract] OR "Allograft Fibrosis"[Title/Abstract]) ("Kidney Transplantation"[Title/Abstract] OR "Renal Transplantation"[Title/Abstract] OR "Kidney Transplant"[Title/Abstract] OR "Renal Transplant"[Title/Abstract] OR "Kidney Allograft"[Title/Abstract] OR "Renal Allograft"[Title/Abstract] OR "Kidney Graft"[Title/Abstract] OR "Renal Graft"[Title/Abstract] OR "Kidney Allograft Transplantation"[Title/Abstract] OR "Kidney Grafting"[Title/Abstract] OR "Renal Grafting"[Title/Abstract] OR "Kidney Retransplantation"[Title/Abstract] OR "Kidney Transplant Recipient"[Title/Abstract] OR "Renal Transplant Recipient"[Title/Abstract] OR "Kidney Recipient"[Title/Abstract] OR "Renal Recipient"[Title/Abstract]) AND ("Air Pollution"[Title/Abstract] OR "Outdoor Air Pollution"[Title/Abstract] OR "Traffic-Related Air Pollution"[Title/Abstract] OR "Air Quality"[Title/Abstract] OR "Polluted Air"[Title/Abstract] OR "Ambient Air Pollution"[Title/Abstract] OR "Atmospheric Pollution"[Title/Abstract] OR "Environmental Pollution"[Title/Abstract] OR "Environmental Exposure"[Title/Abstract] OR "Airborne Pollutants"[Title/Abstract] OR "Environmental Pollutants"[Title/Abstract] OR "Air Pollutants"[Title/Abstract] OR "Particulate Matter"[Title/Abstract] OR "Particulate Air Pollutants"[Title/Abstract] OR "Inhalable Particles"[Title/Abstract] OR "Fine Particulate Matter"[Title/Abstract] OR "Fine Particles"[Title/Abstract] OR "Ultrafine Particles"[Title/Abstract] OR "PM"[Title/Abstract] OR "PM0.1"[Title/Abstract] OR "PM1"[Title/Abstract] OR "PM2.5"[Title/Abstract] OR "PM10"[Title/Abstract] OR "PMcoarse"[Title/Abstract] OR "Coarse Particles"[Title/Abstract] OR "Black Carbon"[Title/Abstract] OR "BC"[Title/Abstract] OR "Ozone"[Title/Abstract] OR "O3"[Title/Abstract] OR "Carbon Monoxide"[Title/Abstract] OR "CO"[Title/Abstract] OR "Nitrogen Oxides"[Title/Abstract] OR "NOx"[Title/Abstract] OR "Nitrogen Dioxide"[Title/Abstract] OR "NO2"[Title/Abstract] OR "Sulfur Dioxide"[Title/Abstract] OR "SO2"[Title/Abstract]) AND ("Graft Survival"[Title/Abstract] OR "Allograft Survival"[Title/Abstract] OR "Kidney Graft Survival"[Title/Abstract] OR "Renal Graft Survival"[Title/Abstract] OR "Transplant Survival"[Title/Abstract] OR "long-term graft survival"[Title/Abstract] OR "short-term graft survival"[Title/Abstract] OR "survival"[Title/Abstract] OR "prognosis"[Title/Abstract] OR "outcome"[Title/Abstract] OR "Death-Censored Graft Failure"[Title/Abstract] OR "DCGF"[Title/Abstract] OR "Graft Failure"[Title/Abstract] OR "Kidney Transplant Failure"[Title/Abstract] OR "Renal Transplant Failure"[Title/Abstract] OR "Graft Loss"[Title/Abstract] OR "Graft Dysfunction"[Title/Abstract] OR "Renal Graft Dysfunction"[Title/Abstract] OR "Kidney Graft Dysfunction"[Title/Abstract] OR "Delayed Graft Function"[Title/Abstract] OR "DGF"[Title/Abstract] OR "Graft Function"[Title/Abstract] OR "Renal Function"[Title/Abstract] OR "Kidney Function"[Title/Abstract] OR "Estimated Glomerular Filtration Rate"[Title/Abstract] OR "eGFR"[Title/Abstract] OR "Glomerular Filtration Rate"[Title/Abstract] OR "GFR"[Title/Abstract] OR "Creatinine Clearance Rate"[Title/Abstract] OR "Serum Creatinine"[Title/Abstract] OR "Plasma Creatinine"[Title/Abstract] OR "Renal Insufficiency"[Title/Abstract] OR "Kidney Insufficiency"[Title/Abstract] OR "Proteinuria"[Title/Abstract] OR "Albuminuria"[Title/Abstract] OR "Urinary Protein-to-Creatinine Ratio"[Title/Abstract] OR "Graft Rejection"[Title/Abstract] OR "Allograft Rejection"[Title/Abstract] OR "Transplant Rejection"[Title/Abstract] OR "Organ Rejection"[Title/Abstract] OR "Acute Rejection"[Title/Abstract] OR "Chronic Rejection"[Title/Abstract] OR "Chronic Allograft Nephropathy"[Title/Abstract] OR "Chronic Antibody-Mediated Rejection"[Title/Abstract] OR "Biopsy-Proven Rejection"[Title/Abstract] OR "BPR"[Title/Abstract] OR "Morbidity"[Title/Abstract] OR "post transplant morbidity"[Title/Abstract] OR "Mortality"[Title/Abstract] OR "All-Cause Mortality"[Title/Abstract] OR "Death"[Title/Abstract] OR "Survival Rate"[Title/Abstract] OR "Infection"[Title/Abstract] OR "Sepsis"[Title/Abstract] OR "Adverse Outcomes"[Title/Abstract] OR "Transplant Complications"[Title/Abstract] OR "Short-Term Outcomes"[Title/Abstract] OR "Long-Term Outcomes"[Title/Abstract] OR "Allograft Fibrosis"[Title/Abstract])  **n=1,137**  **3** "Graft Survival"[Title/Abstract] OR "Allograft Survival"[Title/Abstract] OR "Kidney Graft Survival"[Title/Abstract] OR "Renal Graft Survival"[Title/Abstract] OR "Transplant Survival"[Title/Abstract] OR "long-term graft survival"[Title/Abstract] OR "short-term graft survival"[Title/Abstract] OR "survival"[Title/Abstract] OR "prognosis"[Title/Abstract] OR "outcome"[Title/Abstract] OR "Death-Censored Graft Failure"[Title/Abstract] OR "DCGF"[Title/Abstract] OR "Graft Failure"[Title/Abstract] OR "Kidney Transplant Failure"[Title/Abstract] OR "Renal Transplant Failure"[Title/Abstract] OR "Graft Loss"[Title/Abstract] OR "Graft Dysfunction"[Title/Abstract] OR "Renal Graft Dysfunction"[Title/Abstract] OR "Kidney Graft Dysfunction"[Title/Abstract] OR "Delayed Graft Function"[Title/Abstract] OR "DGF"[Title/Abstract] OR "Graft Function"[Title/Abstract] OR "Renal Function"[Title/Abstract] OR "Kidney Function"[Title/Abstract] OR "Estimated Glomerular Filtration Rate"[Title/Abstract] OR "eGFR"[Title/Abstract] OR "Glomerular Filtration Rate"[Title/Abstract] OR "GFR"[Title/Abstract] OR "Creatinine Clearance Rate"[Title/Abstract] OR "Serum Creatinine"[Title/Abstract] OR "Plasma Creatinine"[Title/Abstract] OR "Renal Insufficiency"[Title/Abstract] OR "Kidney Insufficiency"[Title/Abstract] OR "Proteinuria"[Title/Abstract] OR "Albuminuria"[Title/Abstract] OR "Urinary Protein-to-Creatinine Ratio"[Title/Abstract] OR "Graft Rejection"[Title/Abstract] OR "Allograft Rejection"[Title/Abstract] OR "Transplant Rejection"[Title/Abstract] OR "Organ Rejection"[Title/Abstract] OR "Acute Rejection"[Title/Abstract] OR "Chronic Rejection"[Title/Abstract] OR "Chronic Allograft Nephropathy"[Title/Abstract] OR "Chronic Antibody-Mediated Rejection"[Title/Abstract] OR "Biopsy-Proven Rejection"[Title/Abstract] OR "BPR"[Title/Abstract] OR "Morbidity"[Title/Abstract] OR "Post- Transplant Morbidity"[Title/Abstract] OR "Mortality"[Title/Abstract] OR "All-Cause Mortality"[Title/Abstract] OR "Death"[Title/Abstract] OR "Survival Rate"[Title/Abstract] OR "Infection"[Title/Abstract] OR "Sepsis"[Title/Abstract] OR "Adverse Outcomes"[Title/Abstract] OR "Transplant Complications"[Title/Abstract] OR "Short-Term Outcomes"[Title/Abstract] OR "Long-Term Outcomes"[Title/Abstract] OR "Allograft Fibrosis"[Title/Abstract] "Graft Survival"[Title/Abstract] OR "Allograft Survival"[Title/Abstract] OR "Kidney Graft Survival"[Title/Abstract] OR "Renal Graft Survival"[Title/Abstract] OR "Transplant Survival"[Title/Abstract] OR "long-term graft survival"[Title/Abstract] OR "short-term graft survival"[Title/Abstract] OR "survival"[Title/Abstract] OR "prognosis"[Title/Abstract] OR "outcome"[Title/Abstract] OR "Death-Censored Graft Failure"[Title/Abstract] OR "DCGF"[Title/Abstract] OR "Graft Failure"[Title/Abstract] OR "Kidney Transplant Failure"[Title/Abstract] OR "Renal Transplant Failure"[Title/Abstract] OR "Graft Loss"[Title/Abstract] OR "Graft Dysfunction"[Title/Abstract] OR "Renal Graft Dysfunction"[Title/Abstract] OR "Kidney Graft Dysfunction"[Title/Abstract] OR "Delayed Graft Function"[Title/Abstract] OR "DGF"[Title/Abstract] OR "Graft Function"[Title/Abstract] OR "Renal Function"[Title/Abstract] OR "Kidney Function"[Title/Abstract] OR "Estimated Glomerular Filtration Rate"[Title/Abstract] OR "eGFR"[Title/Abstract] OR "Glomerular Filtration Rate"[Title/Abstract] OR "GFR"[Title/Abstract] OR "Creatinine Clearance Rate"[Title/Abstract] OR "Serum Creatinine"[Title/Abstract] OR "Plasma Creatinine"[Title/Abstract] OR "Renal Insufficiency"[Title/Abstract] OR "Kidney Insufficiency"[Title/Abstract] OR "Proteinuria"[Title/Abstract] OR "Albuminuria"[Title/Abstract] OR "Urinary Protein-to-Creatinine Ratio"[Title/Abstract] OR "Graft Rejection"[Title/Abstract] OR "Allograft Rejection"[Title/Abstract] OR "Transplant Rejection"[Title/Abstract] OR "Organ Rejection"[Title/Abstract] OR "Acute Rejection"[Title/Abstract] OR "Chronic Rejection"[Title/Abstract] OR "Chronic Allograft Nephropathy"[Title/Abstract] OR "Chronic Antibody-Mediated Rejection"[Title/Abstract] OR "Biopsy-Proven Rejection"[Title/Abstract] OR "BPR"[Title/Abstract] OR "Morbidity"[Title/Abstract] OR "post transplant morbidity"[Title/Abstract] OR "Mortality"[Title/Abstract] OR "All-Cause Mortality"[Title/Abstract] OR "Death"[Title/Abstract] OR "Survival Rate"[Title/Abstract] OR "Infection"[Title/Abstract] OR "Sepsis"[Title/Abstract] OR "Adverse Outcomes"[Title/Abstract] OR "Transplant Complications"[Title/Abstract] OR "Short-Term Outcomes"[Title/Abstract] OR "Long-Term Outcomes"[Title/Abstract] OR "Allograft Fibrosis"[Title/Abstract]  **n=5,877,631**  **2** "Air Pollution"[Title/Abstract] OR "Outdoor Air Pollution"[Title/Abstract] OR "Traffic-Related Air Pollution"[Title/Abstract] OR "Air Quality"[Title/Abstract] OR "Polluted Air"[Title/Abstract] OR "Ambient Air Pollution"[Title/Abstract] OR "Atmospheric Pollution"[Title/Abstract] OR "Environmental Pollution"[Title/Abstract] OR "Environmental Exposure"[Title/Abstract] OR "Airborne Pollutants"[Title/Abstract] OR "Environmental Pollutants"[Title/Abstract] OR "Air Pollutants"[Title/Abstract] OR "Particulate Matter"[Title/Abstract] OR "Particulate Air Pollutants"[Title/Abstract] OR "Inhalable Particles"[Title/Abstract] OR "Fine Particulate Matter"[Title/Abstract] OR "Fine Particles"[Title/Abstract] OR "Ultrafine Particles"[Title/Abstract] OR "PM"[Title/Abstract] OR "PM0.1"[Title/Abstract] OR "PM1"[Title/Abstract] OR "PM2.5"[Title/Abstract] OR "PM10"[Title/Abstract] OR "PMcoarse"[Title/Abstract] OR "Coarse Particles"[Title/Abstract] OR "Black Carbon"[Title/Abstract] OR "BC"[Title/Abstract] OR "Ozone"[Title/Abstract] OR "O3"[Title/Abstract] OR "Carbon Monoxide"[Title/Abstract] OR "CO"[Title/Abstract] OR "Nitrogen Oxides"[Title/Abstract] OR "NOx"[Title/Abstract] OR "Nitrogen Dioxide"[Title/Abstract] OR "NO2"[Title/Abstract] OR "Sulfur Dioxide"[Title/Abstract] OR "SO2"[Title/Abstract] "Air Pollution"[Title/Abstract] OR "Outdoor Air Pollution"[Title/Abstract] OR "Traffic-Related Air Pollution"[Title/Abstract] OR "Air Quality"[Title/Abstract] OR "Polluted Air"[Title/Abstract] OR "Ambient Air Pollution"[Title/Abstract] OR "Atmospheric Pollution"[Title/Abstract] OR "Environmental Pollution"[Title/Abstract] OR "Environmental Exposure"[Title/Abstract] OR "Airborne Pollutants"[Title/Abstract] OR "Environmental Pollutants"[Title/Abstract] OR "Air Pollutants"[Title/Abstract] OR "Particulate Matter"[Title/Abstract] OR "Particulate Air Pollutants"[Title/Abstract] OR "Inhalable Particles"[Title/Abstract] OR "Fine Particulate Matter"[Title/Abstract] OR "Fine Particles"[Title/Abstract] OR "Ultrafine Particles"[Title/Abstract] OR "PM"[Title/Abstract] OR "PM0.1"[Title/Abstract] OR "PM1"[Title/Abstract] OR "PM2.5"[Title/Abstract] OR "PM10"[Title/Abstract] OR "PMcoarse"[Title/Abstract] OR "Coarse Particles"[Title/Abstract] OR "Black Carbon"[Title/Abstract] OR "BC"[Title/Abstract] OR "Ozone"[Title/Abstract] OR "O3"[Title/Abstract] OR "Carbon Monoxide"[Title/Abstract] OR "CO"[Title/Abstract] OR "Nitrogen Oxides"[Title/Abstract] OR "NOx"[Title/Abstract] OR "Nitrogen Dioxide"[Title/Abstract] OR "NO2"[Title/Abstract] OR "Sulfur Dioxide"[Title/Abstract] OR "SO2"[Title/Abstract]  **n=1,177,375**  **1** "Kidney Transplantation"[Title/Abstract] OR "Renal Transplantation"[Title/Abstract] OR "Kidney Transplant"[Title/Abstract] OR "Renal Transplant"[Title/Abstract] OR "Kidney Allograft"[Title/Abstract] OR "Renal Allograft"[Title/Abstract] OR "Kidney Graft"[Title/Abstract] OR "Renal Graft"[Title/Abstract] OR "Kidney Allograft Transplantation"[Title/Abstract] OR "Kidney Grafting"[Title/Abstract] OR "Renal Grafting"[Title/Abstract] OR "Kidney Retransplantation"[Title/Abstract] OR "Kidney Transplant Recipient"[Title/Abstract] OR "Renal Transplant Recipient"[Title/Abstract] OR "Kidney Recipient"[Title/Abstract] OR "Renal Recipient"[Title/Abstract] "Kidney Transplantation"[Title/Abstract] OR "Renal Transplantation"[Title/Abstract] OR "Kidney Transplant"[Title/Abstract] OR "Renal Transplant"[Title/Abstract] OR "Kidney Allograft"[Title/Abstract] OR "Renal Allograft"[Title/Abstract] OR "Kidney Graft"[Title/Abstract] OR "Renal Graft"[Title/Abstract] OR "Kidney Allograft Transplantation"[Title/Abstract] OR "Kidney Grafting"[Title/Abstract] OR "Renal Grafting"[Title/Abstract] OR "Kidney Retransplantation"[Title/Abstract] OR "Kidney Transplant Recipient"[Title/Abstract] OR "Renal Transplant Recipient"[Title/Abstract] OR "Kidney Recipient"[Title/Abstract] OR "Renal Recipient"[Title/Abstract]  **n=100,920** |
| **Scopus (n=2208)** |
| TITLE-ABS ( "Kidney Transplantation" OR "Renal Transplantation" OR "Kidney Transplant" OR "Renal Transplant" OR "Kidney Allograft" OR "Renal Allograft" OR "Kidney Graft" OR "Renal Graft" OR "Kidney Allograft Transplantation" OR "Kidney Grafting" OR "Renal Grafting" OR "Kidney Retransplantation" OR "Kidney Transplant Recipient" OR "Renal Transplant Recipient" OR "Kidney Recipient" OR "Renal Recipient" ) AND TITLE-ABS ( "Air Pollution" OR "Outdoor Air Pollution" OR "Traffic-Related Air Pollution" OR "Air Quality" OR "Polluted Air" OR "Ambient Air Pollution" OR "Atmospheric Pollution" OR "Environmental Pollution" OR "Environmental Exposure" OR "Airborne Pollutants" OR "Environmental Pollutants" OR "Air Pollutants" OR "Particulate Matter" OR "Particulate Air Pollutants" OR "Inhalable Particles" OR "Fine Particulate Matter" OR "Fine Particles" OR "Ultrafine Particles" OR "PM" OR "PM0.1" OR "PM1" OR "PM2.5" OR "PM10" OR "PMcoarse" OR "Coarse Particles" OR "Black Carbon" OR "BC" OR "Ozone" OR "O3" OR "Carbon Monoxide" OR "CO" OR "Nitrogen Oxides" OR "NOx" OR "Nitrogen Dioxide" OR "NO2" OR "Sulfur Dioxide" OR "SO2" ) AND TITLE-ABS ( "Graft Survival" OR "Allograft Survival" OR "Kidney Graft Survival" OR "Renal Graft Survival" OR "Transplant Survival" OR "long-term graft survival" OR "short-term graft survival" OR "survival" OR "prognosis" OR "outcome" OR "Death-Censored Graft Failure" OR "DCGF" OR "Graft Failure" OR "Kidney Transplant Failure" OR "Renal Transplant Failure" OR "Graft Loss" OR "Graft Dysfunction" OR "Renal Graft Dysfunction" OR "Kidney Graft Dysfunction" OR "Delayed Graft Function" OR "DGF" OR "Graft Function" OR "Renal Function" OR "Kidney Function" OR "Estimated Glomerular Filtration Rate" OR "eGFR" OR "Glomerular Filtration Rate" OR "GFR" OR "Creatinine Clearance Rate" OR "Serum Creatinine" OR "Plasma Creatinine" OR "Renal Insufficiency" OR "Kidney Insufficiency" OR "Proteinuria" OR "Albuminuria" OR "Urinary Protein-to-Creatinine Ratio" OR "Graft Rejection" OR "Allograft Rejection" OR "Transplant Rejection" OR "Organ Rejection" OR "Acute Rejection" OR "Chronic Rejection" OR "Chronic Allograft Nephropathy" OR "Chronic Antibody-Mediated Rejection" OR "Biopsy-Proven Rejection" OR "BPR" OR "Morbidity" OR "Post- Transplant Morbidity" OR "Mortality" OR "All-Cause Mortality" OR "Death" OR "Survival Rate" OR "Infection" OR "Sepsis" OR "Adverse Outcomes" OR "Transplant Complications" OR "Short-Term Outcomes" OR "Long-Term Outcomes" OR "Allograft Fibrosis" )  **n=2208** |
| **Web of Science (n=1166)** |
| 1: ((TI=("Kidney Transplantation" OR "Renal Transplantation" OR "Kidney Transplant" OR "Renal Transplant" OR "Kidney Allograft" OR "Renal Allograft" OR "Kidney Graft" OR "Renal Graft" OR "Kidney Allograft Transplantation" OR "Kidney Grafting" OR "Renal Grafting" OR "Kidney Retransplantation" OR "Kidney Transplant Recipient" OR "Renal Transplant Recipient" OR "Kidney Recipient" OR "Renal Recipient")) OR AB=("Kidney Transplantation" OR "Renal Transplantation" OR "Kidney Transplant" OR "Renal Transplant" OR "Kidney Allograft" OR "Renal Allograft" OR "Kidney Graft" OR "Renal Graft" OR "Kidney Allograft Transplantation" OR "Kidney Grafting" OR "Renal Grafting" OR "Kidney Retransplantation" OR "Kidney Transplant Recipient" OR "Renal Transplant Recipient" OR "Kidney Recipient" OR "Renal Recipient")) OR AK=("Kidney Transplantation" OR "Renal Transplantation" OR "Kidney Transplant" OR "Renal Transplant" OR "Kidney Allograft" OR "Renal Allograft" OR "Kidney Graft" OR "Renal Graft" OR "Kidney Allograft Transplantation" OR "Kidney Grafting" OR "Renal Grafting" OR "Kidney Retransplantation" OR "Kidney Transplant Recipient" OR "Renal Transplant Recipient" OR "Kidney Recipient" OR "Renal Recipient")  Date Run: Tue Apr 22 2025 12:56:15 GMT+0300 (GMT+03:00)  Results: 132024  2: ((TI=("Air Pollution" OR "Outdoor Air Pollution" OR "Traffic-Related Air Pollution" OR "Air Quality" OR "Polluted Air" OR "Ambient Air Pollution" OR "Atmospheric Pollution" OR "Environmental Pollution" OR "Environmental Exposure" OR "Airborne Pollutants" OR "Environmental Pollutants" OR "Air Pollutants" OR "Particulate Matter" OR "Particulate Air Pollutants" OR "Inhalable Particles" OR "Fine Particulate Matter" OR "Fine Particles" OR "Ultrafine Particles" OR "PM" OR "PM0.1" OR "PM1" OR "PM2.5" OR "PM10" OR "PMcoarse" OR "Coarse Particles" OR "Black Carbon" OR "BC" OR "Ozone" OR "O3" OR "Carbon Monoxide" OR "CO" OR "Nitrogen Oxides" OR "NOx" OR "Nitrogen Dioxide" OR "NO2" OR "Sulfur Dioxide" OR "SO2")) OR AB=("Air Pollution" OR "Outdoor Air Pollution" OR "Traffic-Related Air Pollution" OR "Air Quality" OR "Polluted Air" OR "Ambient Air Pollution" OR "Atmospheric Pollution" OR "Environmental Pollution" OR "Environmental Exposure" OR "Airborne Pollutants" OR "Environmental Pollutants" OR "Air Pollutants" OR "Particulate Matter" OR "Particulate Air Pollutants" OR "Inhalable Particles" OR "Fine Particulate Matter" OR "Fine Particles" OR "Ultrafine Particles" OR "PM" OR "PM0.1" OR "PM1" OR "PM2.5" OR "PM10" OR "PMcoarse" OR "Coarse Particles" OR "Black Carbon" OR "BC" OR "Ozone" OR "O3" OR "Carbon Monoxide" OR "CO" OR "Nitrogen Oxides" OR "NOx" OR "Nitrogen Dioxide" OR "NO2" OR "Sulfur Dioxide" OR "SO2")) OR AK=("Air Pollution" OR "Outdoor Air Pollution" OR "Traffic-Related Air Pollution" OR "Air Quality" OR "Polluted Air" OR "Ambient Air Pollution" OR "Atmospheric Pollution" OR "Environmental Pollution" OR "Environmental Exposure" OR "Airborne Pollutants" OR "Environmental Pollutants" OR "Air Pollutants" OR "Particulate Matter" OR "Particulate Air Pollutants" OR "Inhalable Particles" OR "Fine Particulate Matter" OR "Fine Particles" OR "Ultrafine Particles" OR "PM" OR "PM0.1" OR "PM1" OR "PM2.5" OR "PM10" OR "PMcoarse" OR "Coarse Particles" OR "Black Carbon" OR "BC" OR "Ozone" OR "O3" OR "Carbon Monoxide" OR "CO" OR "Nitrogen Oxides" OR "NOx" OR "Nitrogen Dioxide" OR "NO2" OR "Sulfur Dioxide" OR "SO2")  Date Run: Tue Apr 22 2025 12:56:52 GMT+0300 (GMT+03:00)  Results: 2574340    3: ((TI=("Graft Survival" OR "Allograft Survival" OR "Kidney Graft Survival" OR "Renal Graft Survival" OR "Transplant Survival" OR "long-term graft survival" OR "short-term graft survival" OR "survival" OR "prognosis" OR "outcome" OR "Death-Censored Graft Failure" OR "DCGF" OR "Graft Failure" OR "Kidney Transplant Failure" OR "Renal Transplant Failure" OR "Graft Loss" OR "Graft Dysfunction" OR "Renal Graft Dysfunction" OR "Kidney Graft Dysfunction" OR "Delayed Graft Function" OR "DGF" OR "Graft Function" OR "Renal Function" OR "Kidney Function" OR "Estimated Glomerular Filtration Rate" OR "eGFR" OR "Glomerular Filtration Rate" OR "GFR" OR "Creatinine Clearance Rate" OR "Serum Creatinine" OR "Plasma Creatinine" OR "Renal Insufficiency" OR "Kidney Insufficiency" OR "Proteinuria" OR "Albuminuria" OR "Urinary Protein-to-Creatinine Ratio" OR "Graft Rejection" OR "Allograft Rejection" OR "Transplant Rejection" OR "Organ Rejection" OR "Acute Rejection" OR "Chronic Rejection" OR "Chronic Allograft Nephropathy" OR "Chronic Antibody-Mediated Rejection" OR "Biopsy-Proven Rejection" OR "BPR" OR "Morbidity" OR "Post- Transplant Morbidity" OR "Mortality" OR "All-Cause Mortality" OR "Death" OR "Survival Rate" OR "Infection" OR "Sepsis" OR "Adverse Outcomes" OR "Transplant Complications" OR "Short-Term Outcomes" OR "Long-Term Outcomes" OR "Allograft Fibrosis")) OR AB=("Graft Survival" OR "Allograft Survival" OR "Kidney Graft Survival" OR "Renal Graft Survival" OR "Transplant Survival" OR "long-term graft survival" OR "short-term graft survival" OR "survival" OR "prognosis" OR "outcome" OR "Death-Censored Graft Failure" OR "DCGF" OR "Graft Failure" OR "Kidney Transplant Failure" OR "Renal Transplant Failure" OR "Graft Loss" OR "Graft Dysfunction" OR "Renal Graft Dysfunction" OR "Kidney Graft Dysfunction" OR "Delayed Graft Function" OR "DGF" OR "Graft Function" OR "Renal Function" OR "Kidney Function" OR "Estimated Glomerular Filtration Rate" OR "eGFR" OR "Glomerular Filtration Rate" OR "GFR" OR "Creatinine Clearance Rate" OR "Serum Creatinine" OR "Plasma Creatinine" OR "Renal Insufficiency" OR "Kidney Insufficiency" OR "Proteinuria" OR "Albuminuria" OR "Urinary Protein-to-Creatinine Ratio" OR "Graft Rejection" OR "Allograft Rejection" OR "Transplant Rejection" OR "Organ Rejection" OR "Acute Rejection" OR "Chronic Rejection" OR "Chronic Allograft Nephropathy" OR "Chronic Antibody-Mediated Rejection" OR "Biopsy-Proven Rejection" OR "BPR" OR "Morbidity" OR "Post- Transplant Morbidity" OR "Mortality" OR "All-Cause Mortality" OR "Death" OR "Survival Rate" OR "Infection" OR "Sepsis" OR "Adverse Outcomes" OR "Transplant Complications" OR "Short-Term Outcomes" OR "Long-Term Outcomes" OR "Allograft Fibrosis")) OR AK=("Graft Survival" OR "Allograft Survival" OR "Kidney Graft Survival" OR "Renal Graft Survival" OR "Transplant Survival" OR "long-term graft survival" OR "short-term graft survival" OR "survival" OR "prognosis" OR "outcome" OR "Death-Censored Graft Failure" OR "DCGF" OR "Graft Failure" OR "Kidney Transplant Failure" OR "Renal Transplant Failure" OR "Graft Loss" OR "Graft Dysfunction" OR "Renal Graft Dysfunction" OR "Kidney Graft Dysfunction" OR "Delayed Graft Function" OR "DGF" OR "Graft Function" OR "Renal Function" OR "Kidney Function" OR "Estimated Glomerular Filtration Rate" OR "eGFR" OR "Glomerular Filtration Rate" OR "GFR" OR "Creatinine Clearance Rate" OR "Serum Creatinine" OR "Plasma Creatinine" OR "Renal Insufficiency" OR "Kidney Insufficiency" OR "Proteinuria" OR "Albuminuria" OR "Urinary Protein-to-Creatinine Ratio" OR "Graft Rejection" OR "Allograft Rejection" OR "Transplant Rejection" OR "Organ Rejection" OR "Acute Rejection" OR "Chronic Rejection" OR "Chronic Allograft Nephropathy" OR "Chronic Antibody-Mediated Rejection" OR "Biopsy-Proven Rejection" OR "BPR" OR "Morbidity" OR "Post- Transplant Morbidity" OR "Mortality" OR "All-Cause Mortality" OR "Death" OR "Survival Rate" OR "Infection" OR "Sepsis" OR "Adverse Outcomes" OR "Transplant Complications" OR "Short-Term Outcomes" OR "Long-Term Outcomes" OR "Allograft Fibrosis")  Date Run: Tue Apr 22 2025 12:57:22 GMT+0300 (GMT+03:00)  Results: 6485621    4: #3 AND #2 AND #1  Date Run: Tue Apr 22 2025 12:57:27 GMT+0300 (GMT+03:00)  Results: 1166 |
| **Cochrane Library (n=663)** |
| "Kidney Transplantation" OR "Renal Transplantation" OR "Kidney Transplant" OR "Renal Transplant" OR "Kidney Allograft" OR "Renal Allograft" OR "Kidney Graft" OR "Renal Graft" OR "Kidney Allograft Transplantation" OR "Kidney Grafting" OR "Renal Grafting" OR "Kidney Retransplantation" OR "Kidney Transplant Recipient" OR "Renal Transplant Recipient" OR "Kidney Recipient" OR "Renal Recipient" in Title Abstract Keyword AND "Air Pollution" OR "Outdoor Air Pollution" OR "Traffic-Related Air Pollution" OR "Air Quality" OR "Polluted Air" OR "Ambient Air Pollution" OR "Atmospheric Pollution" OR "Environmental Pollution" OR "Environmental Exposure" OR "Airborne Pollutants" OR "Environmental Pollutants" OR "Air Pollutants" OR "Particulate Matter" OR "Particulate Air Pollutants" OR "Inhalable Particles" OR "Fine Particulate Matter" OR "Fine Particles" OR "Ultrafine Particles" OR "PM" OR "PM0.1" OR "PM1" OR "PM2.5" OR "PM10" OR "PMcoarse" OR "Coarse Particles" OR "Black Carbon" OR "BC" OR "Ozone" OR "O3" OR "Carbon Monoxide" OR "CO" OR "Nitrogen Oxides" OR "NOx" OR "Nitrogen Dioxide" OR "NO2" OR "Sulfur Dioxide" OR "SO2" in Title Abstract Keyword AND "Graft Survival" OR "Allograft Survival" OR "Kidney Graft Survival" OR "Renal Graft Survival" OR "Transplant Survival" OR "long-term graft survival" OR "short-term graft survival" OR "survival" OR "prognosis" OR "outcome" OR "Death-Censored Graft Failure" OR "DCGF" OR "Graft Failure" OR "Kidney Transplant Failure" OR "Renal Transplant Failure" OR "Graft Loss" OR "Graft Dysfunction" OR "Renal Graft Dysfunction" OR "Kidney Graft Dysfunction" OR "Delayed Graft Function" OR "DGF" OR "Graft Function" OR "Renal Function" OR "Kidney Function" OR "Estimated Glomerular Filtration Rate" OR "eGFR" OR "Glomerular Filtration Rate" OR "GFR" OR "Creatinine Clearance Rate" OR "Serum Creatinine" OR "Plasma Creatinine" OR "Renal Insufficiency" OR "Kidney Insufficiency" OR "Proteinuria" OR "Albuminuria" OR "Urinary Protein-to-Creatinine Ratio" OR "Graft Rejection" OR "Allograft Rejection" OR "Transplant Rejection" OR "Organ Rejection" OR "Acute Rejection" OR "Chronic Rejection" OR "Chronic Allograft Nephropathy" OR "Chronic Antibody-Mediated Rejection" OR "Biopsy-Proven Rejection" OR "BPR" OR "Morbidity" OR "Post- Transplant Morbidity" OR "Mortality" OR "All-Cause Mortality" OR "Death" OR "Survival Rate" OR "Infection" OR "Sepsis" OR "Adverse Outcomes" OR "Transplant Complications" OR "Short-Term Outcomes" OR "Long-Term Outcomes" OR "Allograft Fibrosis" in Title Abstract Keyword - (Word variations have been searched)  663 |
| **Ovid Medline (n=1035)** |

**Supplementary Table S2**. Newcastle Ottawa Scale quality assessment of the included studies

| **Study** | **Selection** |  |  |  | **Comparability** | **Outcome** |  |  |  |
| --- | --- | --- | --- | --- | --- | --- | --- | --- | --- |
|  | 1. Representativeness of Exposed Cohort | 2. Selection of Non-Exposed Cohort | 3. Ascertainment of Exposure | 4. Outcome Not Present at Start | 5. Comparability of Cohorts | 6. Assessment of Outcome | 7. Follow-Up Long Enough | 8. Adequacy of Follow-Up | **Total Stars** |
| Feng et al. (2021), (6) | ★ | ★ |  | ★ | ★★ | ★ | ★ |  | 7 |
| Spencer-Hwang et al. (2011), (13) | ★ | ★ |  | ★ | ★ | ★ | ★ |  | 5 |
| Chang et al. (2021) | ★ | ★ |  | ★ | ★★ | ★ | ★ |  | 7 |
| Dehom et al. (2021), (10) | ★ |  |  |  | ★ | ★ | ★ |  | 4 |
| Pierotti et al. (2018), (15) | ★ | ★ |  | ★ | ★ |  | ★ |  | 5 |
| Kim et al. (2021), (14) | ★ | ★ |  | ★ | ★ | ★ | ★ |  | 6 |
| Lin et al. (2020), (6) | ★ | ★ |  | ★ | ★★ | ★ | ★ |  | 7 |
|  |  |  |  |  |  |  |  |  |  |

**Supplementary Table S3.** Definitions of graft rejections for each included studies.

| **Study** | **Definitions** |
| --- | --- |
| **Chang 2021** | Acute kidney rejection reported at 1 year after KT (yes or no) |
| **Dehom 2021, (10)** | N/A |
| **Feng 2021, (6)** | 1-year acute rejection: 1-year acute rejection was defined by having any acute rejection episodes during the first year after KT   - The analysis for 1-year acute rejection was limited to the patients who had follow-up data available during the first year after kidney transplantation. |
| **Kim 2021, (14)** | Rejection was diagnosed based on the criteria established at the 2007 Banff Conference, with biopsy-proven rejections including cases of acute T-cell mediated rejection, acute antibody-mediated rejection, and chronic rejection. |
| **Pierotti 2018, (15)** | N/A |
| **Spencer-Hwang 2011, (13)** | N/A |

**N/A: Not applicable due to the article not examining the variable.**

**Supplementary Table S4. Definitions of cardiovascular outcomes reported in each included study**

| **Study** | **Definitions** |
| --- | --- |
| **Chang 2021, (9)** | N/A |
| **Dehom 2021, (10)** | 1. Total CVD mortality: Defined as any death in which the underlying cause was coronary heart disease (including myocardial infarction or atherosclerotic heart disease), congestive heart failure, cerebrovascular accident (including intracranial hemorrhage or ischemic brain damage/anoxic encephalopathy), cardiac arrhythmia, or cardiac arrest. 2. CHD Mortality: Defined as any death in which the underlying cause was myocardial infarction or atherosclerotic heart disease. 3. CHF Mortality: Defined as any death in which the underlying cause was congestive heart failure. |
| **Feng 2021, (6)** | Cause-specific mortality related to cardiovascular disease, malignancy, and infection were further examined. Mortality data in the SRTR were collected from multiple sources, including follow-up reports submitted by transplant programs, the Centers for Medicare & Medicaid Services End-Stage Kidney Disease (ESKD) Death Notification Form (CMS 2746), and the National Technical Information Service Death Master File. |
| **Kim 2021, (14)** | N/A |
| **Pierotti 2018, (15)** | N/A |
| **Spencer-Hwang 2011, (13)** | The two primary outcomes of interest were death due to CHD and death from natural causes. CHD mortality was defined based on coding within the USRDS database, which has been previously validated. Specifically, deaths were classified as CHD-related if the primary cause of death was recorded as acute myocardial infarction or atherosclerotic heart disease. Natural-cause mortality was defined as death from medical causes, excluding external causes such as accidental injury, suicide, or homicide. |

N/A: Not applicable due to not reporting; CVD: cardiovascular disease; CHD: coronary heart disease; CHF: congestive heart failure.

**Supplementary Table S5.** Population characteristics in the studies analyzed.

| **Study** | **n** | **Mean age (years)** | **Women (%)** | **Dialysis duration (years)** | **Living donor (%)** | **CNI-based IS (%)** |
| --- | --- | --- | --- | --- | --- | --- |
| Moon 2023, (5) | 232 | 46 ± 11 | 39 | — | 100 | — |
| Feng 2021, (6) | 87 233 | 54 (IQR 42 – 63) | 39 | 3.2 (high PM) / 2.3 (low PM) | 34 | 86 |
| Kim 2021, (14) | 1 146 | 45 ± 13 | 40 | 3.8 ± 3.9 | 68 | 93 |
| Dehom 2021, (10) | 1 146 | 50 ± 12 | 38 | 3.5 ± 2.7 | 40 | 90 |
| Spencer-Hwang 2011, (13) | 5 059 | 49 ± 14 | 42 | 3.1 ± 2.5 | 33 | 88 |
| Pierotti 2022, (15) | 6 174 | 48 (IQR 38 – 58) | 40 | 3.0 ± 2.4 | 35 | 87 |

**Supplementary Table S6.** Characteristics of the exposure windows.

| **Study** | **Pre-KT exposure window** | **Post-KT exposure window** | **Metric** |
| --- | --- | --- | --- |
| Moon 2023, (5) | N/A | First post-op year | Annual mean PM₂․₅ |
| Feng 2021, (6) | Calendar year of KT† | Same as pre | Annual mean PM₂․₅ |
| Kim 2021, (14) | Entire dialysis period (mean 3.8 y) | Annual mean thereafter | PM₁₀ |
| Dehom 2021, (10) | 3-yr moving average centred on KT yr | Rolling 3-yr average | PM₂․₅ |
| Spencer-Hwang 2011, (13) | Baseline annual mean | Updated annually | PM₁₀ |
| Pierotti 2022, (15) | Not separated; tied to residential history | Same metric annually | Road-NOx index |

† No separate pre- vs post-exposure; year of KT only.

**Supplementary Table S7.** Cause-specific mortality in the studies screened.

| **Study** | **Exposure comparison** | **Total deaths** | **Cardiovascular deaths (%)** | **Other causes (%)** |
| --- | --- | --- | --- | --- |
| Dehom 2021, (10) | Highest vs lowest PM₂․₅ tertile | 115 | 54 (47 %) | 61 (53 %) |
| Spencer-Hwang 2011, (13) | ≥90th vs ≤10th percentile PM₁₀ | 97 | 37 (38 %) | 60 (62 %) |
| Feng 2021, (6) | Per 10 µg m⁻³ higher PM₂․₅ | 2 402 | 979 (41 %) | 1 423 (59 %) |

**Supplementary Figure S1.** Association between Air Pollution and CHD and CVD Mortality

**
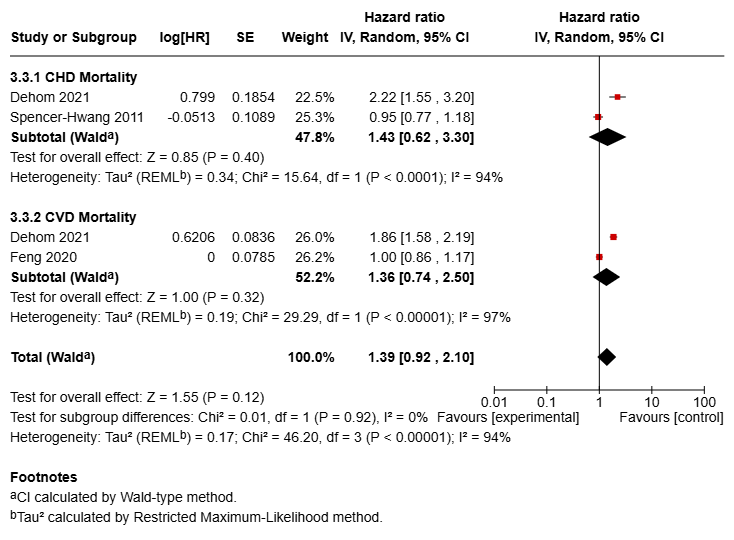
**
